# Emerging Insights into *Corynebacterium Kroppenstedtii* Complex Associated Breast Abscesses: A Retrospective Study

**DOI:** 10.2147/IDR.S501554

**Published:** 2025-05-11

**Authors:** Sreethish Sasi, Manal Mahmoud Mohamed Hamed, Hamad Elnil Abdelgabar Abdel Hadi, Wael Goravey, Emad Ibrahim, Fatma Ben Abid, Sanjay Doiphode, Godwin Wilson, Adila Shaukat Ali Kashaf, Muna Al-Maslamani, Abdullatif Al-Khal

**Affiliations:** 1 Infectious Diseases Division, Department of Medicine, Communicable Diseases Center, Hamad Medical Corporation, Doha, Qatar; 2 Division of Microbiology, Department of Laboratory Medicine and Pathology, Hamad Medical Corporation, Doha, Qatar; 3 Biomedical Research Center, Qatar University, Doha, Qatar; 4 Weill Cornell Medical College, Doha, Qatar; 5 College of Medicine, Qatar University, Doha, Qatar; 6 Infectious Diseases Division, Department of Medicine, Al-Wakra Hospital, Hamad Medical Corporation, Doha, Qatar

**Keywords:** *Corynebacterium kroppenstedtii*, breast-abscess, granulomatous mastitis, hyperprolactinemia, lipophilic bacteria, antimicrobials, microbiological analysis

## Abstract

**Introduction:**

*Corynebacterium kroppenstedtii* complex (CKC), including *C. kroppenstedtii, C. parakroppenstedtii*, and *C. pseudokroppenstedtii*, has been implicated in breast abscesses and granulomatous mastitis, presenting diagnostic and therapeutic challenges. Its lipophilic nature and association with specific populations necessitate targeted investigations.

**Materials and Methods:**

This retrospective study was conducted at Hamad Medical Corporation, Qatar, including all CKC isolates from breast tissue collected between October 2016 and March 2024. Data from electronic medical records were collected and anonymized. Microbiological analysis involved standard culture techniques, Matrix-Assisted Laser Desorption/Ionization Time of Flight Mass Spectrometry (MALDI-TOF MS) for identification, and antibiotic susceptibility testing per Clinical and Laboratory Standards Institute (CLSI) standards. Statistical methods included descriptive analyses, Pearson’s correlation, and relative risk calculations.

**Results:**

Among 34 isolates, 31 were from breast tissue of pre-menopausal women, predominantly obese or overweight, with a median age of 33 years. Breast abscesses were typically unilateral, associated with axillary lymphadenopathy, and varied in size and complexity. Recurrence within six months occurred in 58% of cases. Prolonged antimicrobial therapy and, in some cases, surgical intervention were necessary. Most isolates were sensitive to vancomycin, linezolid, and rifampicin, but resistance to penicillin and daptomycin was noted.

**Conclusion:**

CKC is an emerging pathogen in breast abscesses, requiring precise diagnostic approaches and individualized treatment strategies. Advanced genomic tools are recommended for species differentiation and resistance monitoring. Ongoing research is essential to optimize management and address rising antimicrobial resistance.

## Introduction

The Corynebacterium kroppenstedtii complex (CKC), including *Corynebacterium kroppenstedtii (CK), Corynebacterium parakroppenstedtii (CPK)*, and *Corynebacterium pseudokroppenstedtii*, has emerged as a significant group of pathogens associated with granulomatous lobular mastitis (GLM).^1^ CPK and *C. pseudokroppenstedtii* were recently recognized as distinct species within this complex through genomic and phylogenetic analyses, which revealed their close genetic relationship to CK.^2^ Routine clinical identification methods, such as Matrix-Assisted Laser Desorption/Ionization Time of Flight Mass Spectrometry (MALDI-TOF MS), often fail to differentiate these species, necessitating advanced genomic approaches like 16S rRNA sequencing, whole-genome sequencing, and digital DNA–DNA hybridization for accurate classification.^3^ CPK has been identified as the predominant pathogen in granulomatous lobular mastitis (GLM), associated with higher recurrence rates and elevated prolactin levels, highlighting its unique clinical impact compared to CK.^1–3^ Comparative genomic studies revealed conserved genomic structures, limited divergence, and species-specific virulence factors within CKC, emphasizing the complexity of their identification and their potential roles in GLM pathogenesis.^2^ Improved diagnostic and epidemiological tools, including the identification of core genes, are crucial for understanding and managing CKC infections.^1–3^

This bacterium was first identified from human clinical material in 1998, demonstrating a novel and deep lineage within the genus *Corynebacterium* and distinguished by its lack of mycolic acids, a typical component of the cell wall in many *Corynebacteria*.^4^ The identification and understanding of CKC have evolved with advancements in diagnostic methodologies,^5–8^ highlighting its role in causing granulomatous mastitis, a condition that presents significant diagnostic and therapeutic challenges. The clinical significance of CKC in breast infections came into focus with reports identifying its association with GLM.^4^,^9–12^ The challenges in diagnosing CKC infections stem from its growth requirements and the need for specific culture conditions to ensure its detection.^4^,^5^,^8^ MALDI-TOF MS technology has significantly improved the accuracy of identifying this pathogen, facilitating a more informed approach to managing the associated mastitis.^13^ The prevalence of CKC associated breast abscesses has been explored in various demographic groups, revealing a significant association with psychiatric illnesses, particularly in patients on antipsychotic medications that may induce hyperprolactinemia.^9^,^11^ This connection suggests that drug-induced physiological changes in the breast may predispose certain individuals to infections by this pathogen. Moreover, studies have highlighted the complexity of treating CKC infections, emphasizing the need for prolonged antimicrobial therapy that considers the bacterium’s lipophilic nature and the challenges in achieving effective drug concentrations in the mammary gland.^14^,^15^

In summary, the emergence of CKC as a causative agent of breast abscesses and GLM, and the bacterium’s unique characteristics, including its absence of mycolic acids and its association with specific populations, underline the importance of targeted diagnostic and therapeutic strategies. The evolving insights into the management of CKC infections underscore the need for ongoing research and the adaptation of clinical practices to address the challenges presented by this pathogen effectively.

## Materials and Methods

### Study Design and Setting

This retrospective study was conducted at Hamad Medical Corporation (HMC), the premier provider of tertiary healthcare in Qatar. HMC’s centralized microbiology laboratory which receives samples from all affiliated hospitals (11 tertiary care hospitals with a total bed capacity of 4500) was the primary site of study.

### Study Population

All isolates of CKC from breast tissue, received in HMC’s centralized microbiology laboratory between October 2016 and March 2024 were included in this study. Ethical approval for this study was obtained from the Institutional Review Board of Hamad Medical Corporation (Medical Research Center) under approval numbers MRC-01-22-453, and MRC-01-24-23.

### Data Collection

Patients’ electronic medical records (EMRs) were reviewed to collect demographic data, clinical presentation, underlying health conditions, laboratory and microbiological findings, treatment modalities, and outcomes. Data was anonymized to protect patient confidentiality.

### Microbiological Analysis

Isolation of CKC was performed using standard bacterial culture techniques on blood and chocolate agar plates, incubated at 35–37 °C in 5–10% carbon dioxide (CO2) for 24 to 48 hours. Identification was confirmed by MALDI-TOF MS (Bruker, Billerica, Massachusetts, U.S.A). Antibiotic susceptibility testing was done by ETEST^®^ (bioMérieux, Marcy-étoile, France), and interpreted according to the CLSI performance standards.

### Statistical Analysis

Descriptive statistics were used to summarize the baseline characteristics of the patient cohort. Continuous variables, such as age and duration of symptoms, were reported as medians with interquartile ranges (IQR) or means with standard deviations (SD), depending on their distribution. Categorical variables, such as nationality, comorbidities, and BIRADS stages, were presented as frequencies and percentages. A correlation matrix was constructed to examine the relationship between the size of the abscess and the duration of symptom resolution. Pearson’s correlation coefficient was calculated to determine the strength and direction of the relationship. Two-sample t-tests were employed to compare the mean abscess sizes between different patient groups. Relative risk (RR) was calculated to assess the likelihood of prolonged symptom resolution and extended antibiotic duration among patients with a BMI greater than 25. Prolonged symptom resolution was defined as having active symptoms for more than 3 months, and prolonged antibiotic duration as therapy lasting more than 3 weeks, based on the study’s mean durations. The 95% confidence intervals (CI) were computed to assess the precision of the risk estimates. The statistical significance of findings were determined using p-values. A p-value of less than 0.05 was considered statistically significant. Confidence intervals for relative risks and other estimates were examined to ensure they did not include zero, indicating statistical significance. Statistical analyses were performed using IBM Corp. Released 2017. IBM SPSS Statistics for Windows, Version 25.0. Armonk, NY: IBM Corp.

## Results

### Identification and Case Distribution

**Figure 1:**
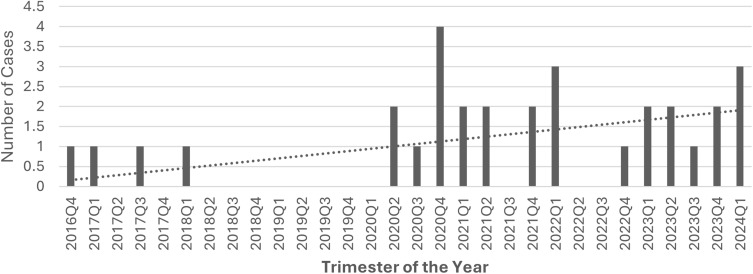
Graphical representation of the increase in the incidence *Corynebacterium kroppenstedtii* complex (CKC) breast abscess cases after January 2020.

34 isolates of CKC were identified during the study period, out of which 31 were from breast tissue. A major rise in the number of cases was observed after January 2020 (87%). Figure 1 shows the 3-monthly distribution of cases from October 2016 to March 2024. The baseline characteristics, clinical features, radiological features, and outcomes of the 31 patients are consolidated in Table 1.

**Table 1:** Baseline Characteristics, Clinical Features, Radiological Features, and Outcomes of All Patients with Corynebacterium Kroppenstedtii Complex (CKC) Breast Abscess (n = 31)

Characteristic				Average (Mean, Median of Mode)
Age				Median 33 (26–46), IQR = 8
Nationality	Filipino			29%
	Qatari			13%
	Indian			16%
	Sri Lankan			9.6%
	Ethiopian			6.5%
	Egyptian			13%
	Palestinian			3%
	Tunisian			3.2%
	Turkey			3.2%
	Sudanese			3.2%
Body Weight				76 (43 −116) kg
BMI			< 18.5	0
			18.5 to 24.9	23.3%
			24.9 to 29.9	30%
			>/= 30	46.6%
Comorbidities			Diabetes Mellitus	0.00%
			Prediabetes	16.6%
			Hypertension	0.00%
			Dyslipidemia	25.81%
			Chronic Kidney Disease	0.00%
			Chronic Liver Disease	0.00%
			Thyroid Disorders	13%; 10% hypothyroid, 3% hyperthyroid
			Solid Organ Tumor	0.00%
			Hematological Malignancy	0.00%
			HIV	0.00%
			Prolactinoma	9.68%
			Use of Dopamine receptor agonists used (Pramipexole, Ropinirole, Bromocriptine, Cabergoline)	9.68%
			Dopamine receptor antagonists used (Antipsychotics, Antiemetics)	0.00%
Laterality			Left	48.3%
			Right	45%
			Bilateral	6.7%
Time to presentation since the last delivery (years)			</= 2	42%
			>2	42%
			Pregnant	6.5%
			No children	9.5%
Lactation in the last 6 months			Yes	16%
Parity			0	9.6%
			1	32.2%
			2	22.5%
			> 2	35.4%
Presenting Complaints			Breast Pain and Swelling	93.5%
			Palpable Mass	55%
			Nipple Discharge	13% (6% serous, 3.5% milky, 3.5% bloody)
Mean duration of Symptoms				40.13 (2–180) days
BIRADS staging			2	31%
			3	48%
			4	21%
Ultrasound findings			1 collection	51.6%
			2 collections	6.9%
			>2 collections	41.5%
Axillary Lymphadenopathy Present				56.6%
Treatment Modalities		Antibiotics		100%
		Steroids		32.2%
		Incision and drainage		22.5%
		Lumpectomy		3.2%
Duration of Antibiotics (In Days)				23.93 (7–74)
Time to complete remission (In weeks)				15 (2 to 52)
Recurrence of symptoms in the next 6 months				58%

### Baseline Characteristics

All were pre-menopausal women with a median age of 33 years (IQR 8). The most represented nationality was Filipino (29%), followed by Indian (16%), Qatari (13%), and Egyptian (13%). Notably, there were no Caucasians affected. 76% of the patients were either overweight or obese according to the World Health Organization’s body mass index (BMI) classifications. Patients with a BMI greater than 25 showed a tendency towards longer symptom resolution (RR = 1.75, 95% CI: 0.30 to 10.16, p = 0.25) and extended antibiotic duration (RR = 1.02, 95% CI: 0.19 to 5.6, p = 1.0), though these findings were not statistically significant.

### Clinical Features

None of the subjects reported smoking or alcohol use. Regarding comorbidities, there was a notable absence of diabetes mellitus and hypertension. 16.6% of patients had pre-diabetes. Mean HbA1c was 5.55. Dyslipidemia was present in 25.81% of the patients, making it the most common comorbidity. Thyroid disorders were observed in 13% of the cohort, with the majority being hypothyroid (10%) and a smaller percentage being hyperthyroid (3%). Other conditions such as chronic kidney disease, chronic liver disease, solid organ tumors, hematological malignancies, psychiatric illnesses, and HIV were not reported. Prolactinoma was present in 9.68% of patients, and the same were using dopamine receptor agonists (bromocriptine, or cabergoline). There were no statistically significant association of the co-morbidities with duration of antibiotic use or the time for resolution of symptoms.

### Presenting Complaints and Radiological Features

The most common presenting complaint was breast pain and swelling, reported by 93.5% of patients, followed by palpable mass in 55%. Nipple discharge was noted in 13% of the patients, categorized as serous (6%), milky (3.5%), or bloody (3.5%). The mean duration of symptoms was 40.13 days, with a wide range from 2 to 180 days. Breast abscesses were almost evenly distributed between the left breast (48.3%) and the right breast (45%), with a smaller percentage of bilateral cases (6.7%). A small proportion of patients (6.5%) were pregnant at the time of presentation, while 9.5% were nulliparous. 42% presented within 2 years of last childbirth, and another 42% after 2 years. 16% of the patients were lactating. The parity distribution revealed that 9.5% had no children, 32.2% had one child, 22.5% had two children, and 35.5% had more than two children.

### BIRADS Staging and Ultrasound Findings

Based on BIRADS staging, 31% of patients were classified as stage 2, 48% as stage 3, and 21% as stage 4. Ultrasound findings varied, with 51.6% showing one collection, 6.9% showing two collections, and 41.5% showing more than two collections. Axillary lymphadenopathy was present in 56.6% of the patients. The average size of the abscesses was 22.14 ± 22.41 (1–97) milliliters. The correlation matrix demonstrated a modest positive correlation of 0.313 between the size of the abscess and the time taken for the resolution of symptoms. This suggests that larger abscesses may be associated with a longer duration for symptom resolution. The *t*-test comparing abscess sizes between patients with and without a recurrent episode in the next six months yielded a t-statistic of 0.009 and a p-value of 0.993. This high p-value indicates that the size of the abscess is not a predictor of recurrence within six months. Conversely, the *t*-test comparing abscess sizes between patients with and without breast pain and swelling showed a t-statistic of −2.614 and a p-value of 0.014. This result is statistically significant, indicating that patients presenting with breast pain and swelling tend to have larger abscesses.

### Histopathology, Treatment, and Outcomes

All patients underwent ultrasound-guided aspiration for tissue diagnosis. Histopathology findings were diverse, with non-necrotizing granulomatous inflammation being the most common (40%), followed by necrotizing granulomatous inflammation (30%). Based on histopathology, 70% had chronic granulomatous mastitis, and 30% acute mastitis. There were no statistically significant correlations between the type of mastitis and any demographic factors or co-morbidities. 74% of the patients were treated with medical therapy alone (antibiotics ± steroids), while 26% underwent early surgical intervention along with antibiotics. There were no statistically significant associations between early surgical intervention and time for resolution of symptoms or recurrence in the next 6 months. All patients received antibiotics as part of their treatment regimen. Steroid use was documented in 32.2% of cases. Surgical interventions included incision and drainage in 22.5% and lumpectomy in 3.2% of patients. The mean duration of antibiotic treatment was 23.93 days, ranging from 7 to 74 days. The time to complete remission of symptoms averaged 15 weeks, with a range from 2 to 52 weeks. Recurrence of symptoms within the next six months was reported in 58% of cases. The most commonly used antibiotic before identification and sensitivity report was amoxicillin-clavulanate and the one after identification and sensitivity report was clindamycin. Table 2 summarises the various antibiotic combinations that were used and the number of patients in whom they were used. There were no statistically significant associations between the combinations of antibiotics used and the occurrence of recurrent episodes within the next 6 months or the time taken for symptom resolution.

**Table 2:** Antibiotic Combinations Used for the Treatment of Patients with Corynebacterium Kroppenstedtii Complex (CKC) Breast Abscesses and the Number of Patients for Whom They Were Used (n=31)

Antibiotic Combination	Frequency	Chi-Square Statistic (Time of Resolution of Symptoms in weeks)	p-value	Chi-Square Statistic Recurrent Episode in the next 6 months)	p-value (Recurrent Episode in the Next 6 months)
Amoxicillin-clavulanate, Clindamycin	10	21.655612	0.707384	12.211111	0.510413
Amoxicillin-clavulanate	6				
Clindamycin	5				
Amoxicillin-clavulanate, Ciprofloxacin	2				
Amoxicillin-clavulanate, Clindamycin, Metronidazole, Cloxacillin	1				
Clindamycin, Cloxacillin	1				
Ciprofloxacin, Clindamycin	1				
Amoxicillin-clavulanate, Doxycycline	1				
Doxycycline	1				
Clarithromycin, Clindamycin	1				
Amoxicillin-clavulanate, Clindamycin, Doxycycline	1				
Amoxicillin-clavulanate, Trimethoprim- Sulfamethoxazole	1				

### Laboratory Findings and Antibiotic Sensitivity

**Figure 2:**
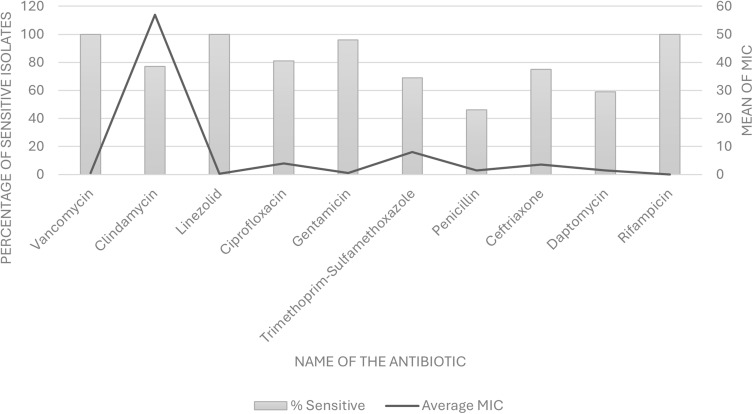
Graphical representation of the percentage of isolates of *Corynebacterium kroppenstedtii* complex (CKC) sensitive to each antibiotic and average minimum inhibitory concentrations (MICs) of each antibiotic.

Laboratory readings of the patients (n = 31) at the time of initial presentation are summarised in Table 3. Figure 2 shows the Minimum Inhibitory Concentrations (MICs) for the antibiotics tested against 31 isolates and the percentage of isolates that were sensitive to each antibiotic, excluding the cases where the test was not available. For each isolate, the MIC was determined by E-test, and susceptibility was determined according to the CLSI guidelines. Vancomycin, linezolid, and rifampicin showed 100% sensitivity among the tested isolates. Less than 50% of the isolates were sensitive to penicillin. Interestingly, daptomycin sensitivity was only 59%.

**Table 3:** Summary of Laboratory Readings of the Patients Effected by Corynebacterium Kroppenstedtii Complex (CKC) Breast Abscesses (n=31)

Parameter	Mean ± Std Dev	Healthy Range	Percentage Outside Healthy Range
CRP (mg/L)	27.99 ± 27.73	0–5 mg/L	83.33%
WBC Count	10.30 ± 3.12	4.0–11.0	44.44%
Hemoglobin (g/dL)	12.42 ± 1.02	12.1–15.1 g/dL	37.04%
Total Cholesterol (mmol/L)	4.78 ± 1.32	< 5.2 mmol/L	23.53%

## Discussion

This retrospective study highlights the significant association between CKC infections and breast abscesses, underscoring the pathogen’s role in GLM. 93% of the isolates of CKC during the study period were identified from breast tissue. Our findings align with previous research, demonstrating the bacterium’s predilection for the mammary glands,^5^,^16–18^ potentially due to its lipophilic nature. Our analysis revealed a rapid increase in the number of cases after January 2020. This shows that CKC is emerging as a leading cause of breast abscesses as shown in latest literature.^17^,^19^,^20^ Identification of CKC has evolved with advances in diagnostic methodologies, including MALDI-TOF MS,^13^ enabling more accurate detection and understanding of its pathogenicity. Our study re-affirms the lower predilection of Caucasians to CKC breast abscess, as shown in literature.^12^,^21^ Absence of traditional risk factors like diabetes mellitus in our cohort suggests that other, less understood factors may predispose individuals to these infections. Further complicating the clinical management of CKC infections is the pathogen’s ability to cause recurrent infections.^17^,^21–23^ The recurrent nature of breast abscesses due to CKC, despite surgical intervention and antibiotic treatment, suggests an intricate interaction between the pathogen and host immune responses. The case of a young Asian female experiencing recurrent abscesses despite drainage and antibiotics exemplifies this challenge, highlighting the necessity for tailored therapeutic approaches that consider both microbial factors and host immune status.^24^

The successful treatment of GLM associated with CKC, as shown in several case reports, involves prolonged antimicrobial therapy considering the bacterium’s characteristics. Waldron et al^14^ reported successful treatment with clarithromycin and rifampin over three months, with no recurrence after 16 months, highlighting the importance of prolonged and appropriate antibiotic therapy. Figure 2 shows that over the years the organism has been slowly acquiring resistance to multiple antibiotics such as penicillin, ceftriaxone, trimethoprim-sulfamethoxazole, clindamycin and daptomycin. The characterization of CKC’s resistance profiles, as demonstrated in the case reports and genomic studies, points to an intriguing aspect of its biology.^5^,^25^ The discovery of a specific genomic island in the multidrug-resistant isolate CNM633/14, akin to the R plasmid of *Corynebacterium resistens*, suggests horizontal gene transfer might play a pivotal role in its pathogenesis and resistance mechanisms^26^. This genomic adaptability could explain the clinical observation of variable response to antibiotic therapy and underscores the importance of genomic surveillance in understanding and combating this pathogen’s spread and treatment resistance.

Our study’s findings on the correlation between abscess size, symptom duration, and treatment outcomes further contribute to the literature, providing insights into managing this challenging condition. Current data suggests that treatment strategies involving longer courses of antibiotics, as well as the use of steroids, might be associated with longer recovery periods and a higher chance of recurrent infection episodes.^12^,^14^,^15^ This could reflect more severe initial infections or a more cautious approach by clinicians in cases perceived as more complex or at higher risk of complications. The choice of treatment approach, including the decision to use surgical intervention alongside medical treatment, appears to influence the duration of antibiotic therapy, which in turn, may affect patient outcomes such as the time to symptom resolution and the likelihood of recurrence.^12^,^16^ However, our study failed to show any statistically significant association between duration or choice of antibiotics, use of steroids, early surgical intervention, and outcomes. This analysis highlights the importance of personalizing the duration and type of antibiotic therapy, the use of steroids, and the choice between surgical and medical treatment in managing CKC associated breast abscesses. Further research could explore the mechanisms behind these correlations and develop optimized treatment protocols to improve patient outcomes.

The association between CKC infection and hyperprolactinemia, particularly in patients with psychiatric illnesses on antipsychotic medication,^11^ underscores a complex interplay between physiological changes and susceptibility to infection. The observation that drug-induced hyperprolactinemia may predispose individuals to these infections points to the need for careful management of patients on long-term antipsychotic therapy, emphasizing the role of interdisciplinary care in preventing and managing such infections. In our study, prolactinoma and hyperprolactinemia were present in 9.68% of patients, and the same were using dopamine receptor agonists (bromocriptine, or cabergoline). However, none of the patients had a history of psychiatric illness or were using antipsychotic medications.

Adding to the complexity is the impact of diagnostic challenges on treatment outcomes. The difficulty in isolating and accurately identifying CKC, as well as determining its antibiotic susceptibility, can delay appropriate treatment, potentially leading to worse outcomes. The successful identification and treatment of two cases of GLM in nulliparous young women with hyperprolactinemia^9^ highlight the critical role of advanced molecular techniques, such as 16S rRNA gene sequencing, in the accurate diagnosis and effective management of this condition. Lastly, the varied antibiotic susceptibility profiles observed across different isolates of CKC necessitate a personalized approach to antibiotic therapy. The use of antibiotics with high lipid solubility, such as rifampin and clarithromycin, has shown promise in treating infections caused by this pathogen,^14^ reflecting its lipophilic nature and the necessity for targeted antimicrobial strategies. This approach aligns with our study’s emphasis on prolonged antimicrobial therapy and highlights the importance of considering the bacterium’s unique characteristics in treatment planning.

## Limitations

The sample size is relatively small, and larger studies would be needed to confirm these trends and further investigate the complex dynamics between treatment approaches and patient outcomes. The identification of CKC isolates in this study relied on MALDI-TOF MS, which, while effective, has limitations in distinguishing closely related species like CK, CPK, and *C. pseudokroppenstedtii*. Advanced genomic methods, such as 16S rRNA sequencing and whole-genome analysis, are needed for precise species identification within CKC. Additionally, comparative genomic studies highlight the conserved structure and limited divergence among CKC species, making single-gene identification approaches less effective. Future research should incorporate comprehensive genomic tools to provide deeper insights into the taxonomy, pathogenicity, and resistance mechanisms of CKC species, addressing these critical gaps in our understanding.

## Conclusion

This retrospective study highlights the importance of CKC as a significant pathogen in breast abscesses and GLM. The complexity of diagnosing and treating infections caused by this bacterium calls for further research into its pathogenic mechanisms, resistance patterns, and optimal management strategies. Advanced diagnostic techniques and tailored therapeutic approaches can improve outcomes for patients affected by this challenging pathogen. The study adds to the growing body of evidence suggesting that obesity and dyslipidemia may predispose individuals to breast infection by this pathogen. Successful management of these infections relies on accurate identification and prolonged antimicrobial therapy tailored to the bacterium’s unique characteristics. Future research should focus on elucidating the mechanisms underlying CKC’s pathogenicity and the factors contributing to its association with specific populations.

## Data Availability

Data supporting the conclusions of the study is all available free of cost through open access journals and websites.

## Abbreviations

CKC, *Corynebacterium kroppenstedtii* complex; CK, *Corynebacterium kroppenstedtii*; CPK,* Corynebacterium parakroppenstedtii*; GLM, granulomatous lobular mastitis; MALDI-TOF MS, Matrix-Assisted Laser Desorption/Ionization Time of Flight Mass Spectrometry; rRNA, ribosomal ribo-nucleic acid; EMR, Electronic Medical Record; HMC, Hamad Medical Corporation; BMI, Body Mass Index; CRP, C-Reactive Protein; WBC, White Blood Cell; MIC, Minimum Inhibitory Concentration; RR, Relative Risk; CI, Confidence Interval; CLSI, Clinical and Laboratory Standards Institute.
